# Research on Distinguishing Fish Meal Quality Using Different Characteristic Parameters Based on Electronic Nose Technology

**DOI:** 10.3390/s19092146

**Published:** 2019-05-09

**Authors:** Pei Li, Zouhong Ren, Kaiyi Shao, Hequn Tan, Zhiyou Niu

**Affiliations:** 1 College of Engineering, Huazhong Agricultural University, Wuhan 430070, China; huanonglipei8@163.com (P.L.); renzou_hong@163.com (Z.R.); shao_kaiyi@163.com (K.S.); thq@mail.hzau.edu.cn (H.T.); 2 Key Laboratory of Agricultural Equipment in Mid-lower Yangtze River, Ministry of Agriculture, Wuhan 430070, China

**Keywords:** electronic nose, fish meal, characteristic parameters, PCA, LDA, classifier

## Abstract

In this paper, a portable electronic nose, that was independently developed, was employed to detect and classify a fish meal of different qualities. SPME-GC-MS (solid phase microextraction gas chromatography mass spectrometry) analysis of fish meal was presented. Due to the large amount of data of the original features detected by the electronic nose, a reasonable selection of the original features was necessary before processing, so as to reduce the dimension. The integral value, wavelet energy value, maximum gradient value, average differential value, relation steady-state response average value and variance value were selected as six different characteristic parameters, to study fish meal samples with different storage time grades. Principal Component Analysis (PCA) and Linear Discriminant Analysis (LDA), and five recognition modes, which included the multilayer perceptron neural network classification method, random forest classification method, k nearest neighbor algorithm, support vector machine algorithm, and Bayesian classification method, were employed for the classification. The result showed that the RF classification method had the highest accuracy rate for the classification algorithm. The highest accuracy rate for distinguishing fish meal samples with different qualities was achieved using the integral value, stable value, and average differential value. The lowest accuracy rate for distinguishing fish meal samples with different qualities was achieved using the maximum gradient value. This finding shows that the electronic nose can identify fish meal samples with different storage times.

## 1. Introduction

Fish meal is a high protein feed made from one or more kinds of fish, which is deoiled, dehydrated, and crushed. With an increase in the aquaculture scale in China in recent years, the demand for aquatic feed is rapidly increasing, and the demand for fish meal shows sustained growth. Fish meal is a highly valued source of feed proteins because it is easily digested and has excellent essential amino acids, other essential nutrients, and DHA. Fish meal is an excellent source of vitamins (such as riboflavin, nicotinic acid, vitamin A, and vitamin D) and minerals (such as calcium, phosphorus, iron, zinc, selenium, and iodine) [1] and is the main animal-derived feed raw material in the feed industry. Therefore, the quality of fish meal directly affects the quality of feed products [2]. During the storage process of fish meal, the quality and nutritional components of fish meal vary by different degrees due to weather changes, temperature, humidity, mold, and other microorganisms, which affect the digestion and immunity of animals [3,4]. Therefore, the detection of fish meal quality is an important technical link and means to ensure the quality of feed products.

In case of odor detection, the traditional instruments, such as gas chromatography (GC), gas chromatography/chemiluminescence (GC/SCD), and gas chromatography/mass spectrometry (GC/MS) are mostly employed [5,6]. Conventional physical and chemical analysis methods, such as chemical detection, sensory evaluation, and spectral analysis [7] are also employed. Unfortunately, they are destructive and time consuming for sample measurement. Furthermore, these instruments are very expensive and bulky.

Bionic olfaction technology is a new detection technology that has been developed in recent years. Currently, this technology is prevalent. Some scholars have applied this technology to the quality inspection of agricultural products, including the quality inspection of food crops [8,9,10,11,12], fruit [13,14,15,16,17], meat [18], and other plant crops [19,20,21], and the discrimination is satisfactory. Ghasemi-Varnamkhasti M [22] et al. applied bionic olfaction technology to detect beer aging; Natale C D [23,24] et al. applied this technology to detect the freshness of fish. Gobbi E [25,26,27,28] et al. applied bionic olfaction technology to colony detection, with high accuracy; Lim J H [29,30] et al. applied this technology to medical research. Therefore, the electronic nose has potential in detecting fish meal quality. Although many classification and prediction techniques, such as principal component analysis (PCA), support vector machines (SVM), are employed to process nonlinear measurement data from E-nose systems, the application in fish meal is still necessary.

No information is available in the application of an E-nose in assessing fish meal quality. In the quality assessment by an E-nose, feature extraction and selection are of great importance. Because of large quantities of data, it would be more useful to segregate the irrelevant or redundant attributes from relevant and important ones, although the exact governing rules may not be known. Feature selection provides a means for choosing the features that were best for classification, based on various criteria. Many feature extraction methods have been reported, while none is relevant with E-nose feature vectors extraction, which could sufficiently represent the information of the fish meal quality. Most of the features selected in the literature are the integral value, but the integral value can only reflect the overall information of the sample, and cannot reflect the dynamic information, that is, the mainstream information of the sample. The objectives of this research are to determine whether it is possible to distinguish fish meal with different storage times by an E-nose, and whether the feature vectors chosen are effective in the discrimination of fish meal samples, with different storage times. So the original features should be reasonably selected, while retaining as much information as possible.

In this work, we have designed and constructed a portable and low-cost E-nose, based on ten gas sensors to assess the freshness fish meal stored at different storage times. The integral value, average differential value, and relation steady-state response average value of sensor response curve were often used to characterize sample information [31]. In addition, the energy value, maximum gradient value, and variance value were employed as characteristic parameters to distinguish fish meal samples with different storage times. Based on these characteristic values, this study investigated fish meal samples of different qualities, determined appropriate characteristic values, and achieved better distinguishing results for fish meals of different qualities by some classification algorithms, such as PCA, LDA.

## 2. Materials and Methods

### 2.1. Test Material

Fresh fish meals, which were obtained from a feed company in hubei province, were placed in a thermostatic artificial climate box (RGX-250B, Shanghai Kuntian Instrument Co., Ltd., Shanghai, China) at 35 °C to make the fish meals gradually deteriorate, with an extended storage time. The fish meals were collected as test samples during storage with different storage times. A total of 6 fish meal samples of storage grades were selected for data collection of the detection device. The 6 fish meal samples were classified according to the acid value, which was made as a freshness evaluation index. The acid value of each grade sample was shown in the Table 1.

### 2.2. Bionic Olfaction Detection System

**Figure 1:**
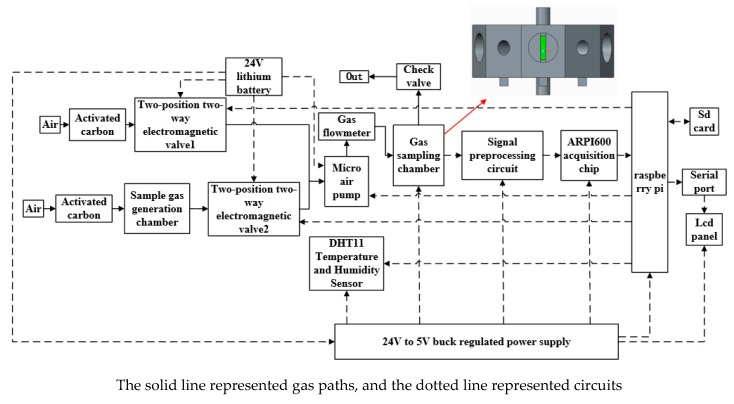
Block diagram of detection device system.

The system employed in the experiment was a portable fish meal quality detection system, based on bionic olfaction. As shown in Figure 1, the system primarily consisted of four parts; (I) a gas acquisition and transmission module; (II) a control processing storage module with strawberry pie as the core; (III) an ARPI600 data acquisition module; and (IV) a sensor array module. The sensor array module was the core component of the device and primarily consisted of 10 MOS (metal oxide semiconductor) gas sensors. The models of each sensor, and the gases that can be measured, are shown in Table 2. The ten gas sensors were selected in order to cover the volatile organic compounds in fish meal.

Gas sensors had broad spectrum response characteristics and can respond to a variety of gas components. This characteristic enabled the constructed array to test a variety of samples. The loop measurement voltage of the gas sensor array was (5 ± 0.2) V, and the heating voltage was (5 ± 0.2) V. In addition, the electronic nose was equipped with temperature and humidity sensors to measure the environmental temperature and humidity information to compensate the influence of the measuring environment on the gas sensor. The temperature and humidity sensors were integrated components (Guangzhou Aosong Electronics Co., Ltd., Guangzhou, China). The model is DHT11, and the measuring voltage is (5 ± 0.01) V. The temperature measuring range was 0~50 °C, the relative humidity measuring range was 20%~90%.

The key component of the device was the self-designed sensor sampling chamber, which consisted of an upper shell, a center body, and a lower shell. The shape of the upper shell with an air outlet was a regular prism; the first mounting cavity had the shape of a regular prism, and mounting holes were formed in the center of each side surface. The number of sensors was the same as the number of the side surfaces of the upper shell; each sensor was mounted in each mounting hole. The shape of the central body was a regular prism; the size of the central body matched the size of the first mounting cavity; a plurality of gas channels were uniformly arranged along the surface of the central body, and the number of the gas channels was equal to the number of the side surfaces of the central body. An air inlet was arranged in the center of the lower shell. This ensured the synchronization and uniformity of the detection of sample gas by each sensor. At the same time, it also ensured the full contact between the sample gas and the sensor, and reduced the response and recovery time. The volume of the gas sampling chamber is only 0.059 L, which effectively reduced the volume. The material used in the gas sampling chamber is polytetrafluoroethylene (PTFE) that has the characteristics of thermo-stability, slushing, not easily bonding, and good sealing. It is suitable for manufacturing the gas sampling chamber.

First, the detection device was pre-heated with electricity for half an hour to eliminate the any instability in the instrument. In this experiment, the gas flow rate was set at 2.2 L·min^−1^. Then, the valve 1 was opened and the valve 2 was closed, the device was cleaned with purified air that was filtered by activated carbon for 77 s. Last, the fish meal sample (80 g) was placed in a 250 mL high borosilicate headspace vials; the valve 2 was opened and the valve 1 was closed, the top air generated by the sample was pumped into the gas sampling chamber of the detection device by a micro air pump and chemically reacts with the gas sensor located in the sampling chamber, which caused the resistance of the sensor to change. The detection time of the device was 39 s and the data sampling interval was 1 s. After each sampling, the device needed to be cleaned and restored to test the next sample. All device controls were processed using qt creator programming. All the parameters selected above were optimized. 

### 2.3. SPME-GC-MS Analysis

Accurately weigh the sample 3 ± 0.1 g in a 20 mL headspace vials, add a certain amount of saturated NaCl solution (10 mL), put it in a constant temperature magnetic stirrer and balance it [32]. After 10 min equilibration at 50 °C, SPME fiber (50/30 µm DVB/CAR/PDMS by Supelco) was exposed for 30 min in the vial headspace. Then, the fiber was immediately desorbed for 5 min in a gas chromatograph injection port operating in split mode with split ratio of 5:1 at 250 °C. 

The identification of volatile compounds was performed by gas chromatograph (Thermo Fisher Scientific) coupled to ISQ mass spectrometry using a Trace GC-MS Thermo Fisher Scientific, equipped with a TG-5MS chromatographic column (30 m × 0.25 mm with 0.25 µm film thickness). Initial column temperature was held at 40 °C for 5 min, and then increased to 120 °C at 4 °C/min, with hold time of 1 min, and finally to 250 °C at 13 °C/min, with hold time of 5 min. Helium was used as the carrier gas at 1 mL/min constant flow. The temperature of ion source and transfer line was 230 °C, and 280 °C, respectively. The mass detector was operated in full scanning mode in the range between *m/z* 50–500, with IE energy of 70 eV. Chromatograms and spectra were recorded and processed using Xcalibur software (Thermo Fisher Scientific).

Compounds were identified by comparison of their mass spectra and retention times with those of injected authentic standards (C7–C40) [33]. 

RI (Retention index) was calculated as follows under single linear programming warming conditions:

(1) $$RI=100n+100\times \frac{T_{Rx}-T_{Rn}}{T_{Rn+1}-T_{Rn}}$$

In the formula: *T_Rx_* is the retention time of the components to be measured; *n* and *n* + 1 represent the number of carbon atoms of n-alkanes, respectively; *T_Rn_* < *T_Rx_* < *T_Rn_*_+1_.

### 2.4. Experiment Design and Data Processing

The fish meal samples were measured using E-nose, based on the optimum experiment conditions. A total of 6 fish meal samples of storage grades were measured, and every sample was tested 30 times in parallel. Based We calculated the relative standard deviation (RSD, n = 30) of the relation steady-state response average value for each sensor.

Savitzky-Golay was adopted to perform five-point filtering and smoothing processing [34], in order to eliminate the noise signals. Six features were extracted, including integral value (INV), wavelet energy value (WEV), maximum gradient value (MGV), average differential value (ADV), relation steady-state response average value (RSAV), and variance value (VARV), as characteristic parameters of the electronic nose signals of fish meal samples. A total of 10 × 6 characteristic parameters were extracted to form a 60-dimensional characteristic matrix. Because the dimensions of the six eigenvalues differed, the data of each eigenvalue must be normalized. In this paper, normalization was utilized to normalize the data, as shown in the formula (1). PCA and LDA analysis methods were employed to analyze the classification results of each feature. A multi-layer perceptron neural network [35,36,37], random forest [38], K-nearest neighbor (K = 3) [39], support vector machine [40] and Naïve Bayes [41] classification methods were simultaneously employed to classify fish meal of different qualities with 6 characteristic values as inputs, and the classification accuracy rate for each characteristic value was obtained.

(2) $$x_{i}^{\prime }=\frac{x_{i}-x_{\min}}{x_{\max}-x_{\min}}$$

where *x′_i_* is the normalized characteristic value; *x_i_* is the eigenvalue of the original data; *x_max_* is the maximum value of the original feature; and *x_min_* is the minimum value of original features.

## 3. Results and Discussion

### 3.1. Application of Electronic Nose to the Odor of Fish Meal of Different Qualities

#### 3.1.1. Repeatability of E-Nose Experiment

As shown in Table 3, the RSD values of all sensors response to the sample Samples numbered 1 were less than 5%, indicating that the experiment has good repeatability, that is, the sensors did not exhibit a “memory” to prior exposure sample, cleaning in fresh air always brought the sensors back to approximately same resistance as initial. Therefore, it was found that the use of E-nose in detecting fish meal odor resulted in good repeatability, sensitivity, effectiveness, and stability.

#### 3.1.2. Response of Electronic Nose to Fish Meal of Different Qualities

**Figure 2:**
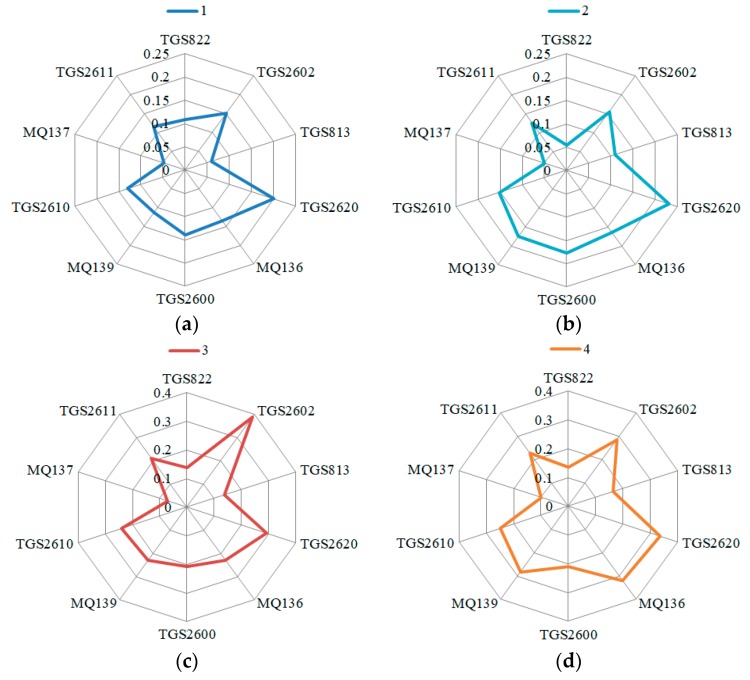

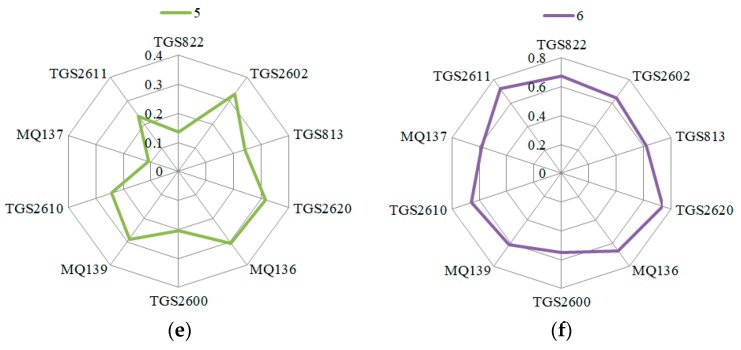
Fingerprints of different fish meal samples with the relation steady-state response average value as an example (samples numbered 1 (**a**); samples numbered 2 (**b**); samples numbered 3 (**c**); samples numbered 4 (**d**); samples numbered 5 (**e**); samples numbered 6 (**f**)).

Figure 2 was the fingerprint of fish meal of different qualities obtained by considering the relation steady-state response average value, as an example. As shown in the figure, the fingerprint of fish meal of different qualities differed to some extent, which provided the basis for the classification of fish meal quality in the future. Therefore, in subsequent research, the impact of each sensor’s characteristic values on the classification of fish meal samples of different qualities must be analyzed to compare the contribution of different characteristic values. 

Based on gas chromatography-mass spectrometry, more than 30 compounds, including aldehydes, alcohols, ketones, nitrogen containing, oxygen containing or sulphur-containing compounds, esters, and alkanes were identified in fish meal. These volatile organic compounds (VOCs) contain both reducing and oxidizing agents that increase/decrease the electrons from crystal surface of MOS (Metal oxide semiconductor), leading to the decrease/increase in resistance of gas sensor. Therefore, a little decrease in sensor responses may cause from suppression of oxidizing gas emission. In addition, the TGS 2620 and TGS 2602 show high sensitivity in sensor responses with different fish meal samples. In fact, TGS 2620 and TGS 2602 exhibit high sensitivity to alcohols. In case of the fish meal, alcohols were found to be one of the most dominant volatiles in the fish meal. It suggests that alcohol odors may play an important role in detection of fish meal freshness based on single gas sensor. With the increase in storage levels, the response of MQ137 gradually increased, indicating that the concentration of ammonia and amine compounds increased gradually with the increase of storage time. In sample 6, the response of MQ137 increased rapidly, indicating severe corrupt samples had higher concentrations of ammonia and amines which were found in GC-MS assays. To support our above proposed sensing mechanism, the volatile compounds of fish meal were analyzed by using GC/MS. All volatile compounds of fish meal are listed in Table 4. One can be observed from Table 4 that no H_2_S was found from GC-MS experiment but MQ136 showed a response to fish meal (see Figure 2). This comes from cross sensitivities of MQ136 to many VOCs such as ethanol, ammonia, and toluene.

### 3.2. Feature Extraction and Selection

The selection of characteristic parameters of gas-sensitive signals has a considerable influence on the accuracy of the detection model. In this paper, six eigenvectors were selected to represent fish meal samples with different qualities via response curves. These eigenvalues were the integral value, wavelet energy value, maximum gradient value, average differential value, relation steady-state response average value, and variance value.

#### 3.2.1. Integral Value

Integral value (INV): The integral value was the area between the sensor response signal curve and its baseline; it reflected the total response result of the sensor to the volatile components of the tested samples. The calculation formula was expressed as follows:

(3) $$INV=\sum_{i=1}^{T}X_{i}\Delta t$$

In the formula, *X_i_* is the response value of the sensor to the ith second of a sample; Δt is the time interval between two adjacent sampling points, 1 s is selected; and *T* is the acquisition time of a sample by the sensor.

#### 3.2.2. Wavelet Energy Value

Wavelet energy value (WEV): The db3 wavelet function was used to perform 4-layer wavelet decomposition with the original data of the sensors. Each sensor obtained 7 approximate coefficients as its characteristics, and the sum of squares of each coefficient was considered as the energy value of each sensor.

(4) $$WEV=\sum_{y=1}^{m}\alpha_{4y}^{2}$$

where α_4*y*_ is the yth decomposition coefficient in the set of approximation coefficients after four-scale decomposition of the signal, and m is the total number of coefficients in the set of approximation coefficients.

#### 3.2.3. Maximum Gradient Value

Maximum gradient value (MGV): The response curve of the gas sensor had a maximum response value. Thus, the slope of the line segment between the maximum value and the initial value could be used to express the signal change speed in the initial stage.

(5) $$MGV=\frac{x_{i\max}-x_{0}}{t}$$

where *x_imax_* is the maximum response value that corresponds to a sample; *x*_0_ is the initial response value of a sample; and *t* is the response time that corresponds to the maximum response value.

#### 3.2.4. Average Differential Value

Average differential value (ADV): The average differential value was a method that could comprehensively reflect the total information of the sensor’s dynamic response process and directly reflected the mainstream information of the gas sensor’s response to gas.

(6) $$ADV=\frac{1}{T-1}\sum_{i=1}^{T-1}\frac{x_{i+1}-x_{i}}{\Delta t}$$

In the formula, *x_i_* is the response value of the sensor to the ith second of a sample; Δt is the time interval between two adjacent sampling points, 1 s; and *T* is the acquisition time of a sample by the sensor.

#### 3.2.5. Relation Steady-State Response Average Value

Relation steady-state response average value (RSAV): The response curve of the gas sensor had a relatively stable interval; thus, the average value of this interval could be used to characterize the stable characteristic. The calculation formula was expressed as follows:

(7) $$RSAV=\frac{\sum_{i=t_{0}}^{T}x_{i}}{T-t_{0}}$$

In the formula, *x_i_* is the response value of the sensor to the ith second of a sample; *t*_0_ is the corresponding time when the stability is to be reached; and *T* is the acquisition time of a sample by the sensor.

#### 3.2.6. Variance Value

Variance value (VARV): Variance reflected the degree of data dispersion, and the response signal variance was used to represent the characteristic value of its signal strength.

(8) $$VARV=\frac{\sum_{i=1}^{N}{\left(x_{i}-\bar{x}\right)}^{2}}{n}$$

where $\bar{x}$ is the mean value of the sensor response signal to a sample, *x_i_* is the response value of the sensor to the ith second of a sample, and *n* is the time points of the sensor

### 3.3. Principal Component Analysis (PCA)

**Figure 3:**
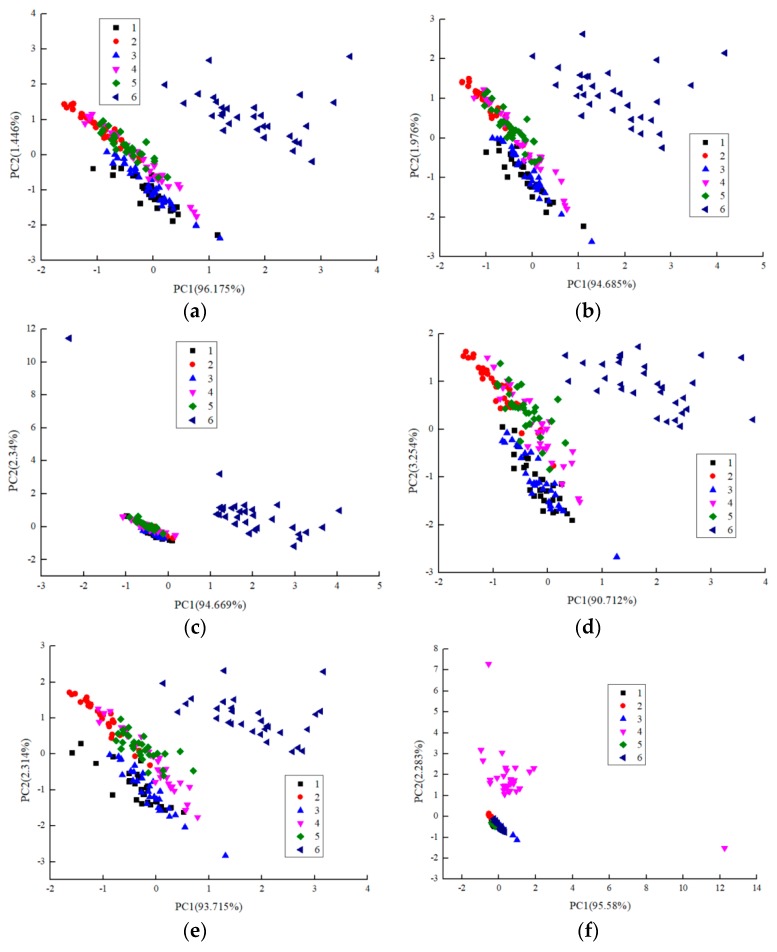
Principal Component Analysis (PCA) analysis results for different characteristic values (integral value (**a**); wavelet energy value (**b**); maximum gradient value (**c**); average differential value (**d**); relation steady-state response average value (**e**); and variance value (**f**)).

PCA could deduct dimensions and observe a primary evaluation of the between-class similarity PCA is a projection method that allows an easy visualization of all the information contained in a data-set. In addition, PCA helps to find out in what respect a sample is different from others and which variables contribute most to this difference. The integral value, wavelet energy value, maximum gradient value, average differential value, relation steady-state response average value and variance value were applied as characteristic parameters to perform a principal component analysis on fish meal at six different storage time levels. Figure 3 showed a principal component analysis diagram of fish meal of six different qualities with different characteristic parameters. The diagram showed that the sum of the first principal component analysis value and the second principal component analysis value was greater than 85% when all characteristic values were employed as parameters. This finding showed that the electronic nose adequately reflected the volatile gases of fish meal at different storage times, and the distribution results were similar but the classification results were not satisfactory. For each feature vector classification result, all fish meal samples were divided into two categories—the sixth and the other—that is, rotten samples were clearly distinguished from fresh samples but the distinction was almost not distinct for the first five categories of samples, which indicated that the odor volatilized from rotten samples and fresh samples distinctly differed while all fresh samples overlapped due to similar volatile odors. Considering all PCA analysis charts, when the relation steady-state response average value was employed as the characteristic parameter of fish meal of different quality grades, the PCA analysis chart had a better discrimination effect, and the discrimination effect using the maximum gradient value and the variance value was the worst. In some cases, the distance between groups was smaller (fresh samples) and the distance within groups was larger (corrupt samples).

### 3.4. Linear Discriminant Analysis (LDA)

**Figure 4:**
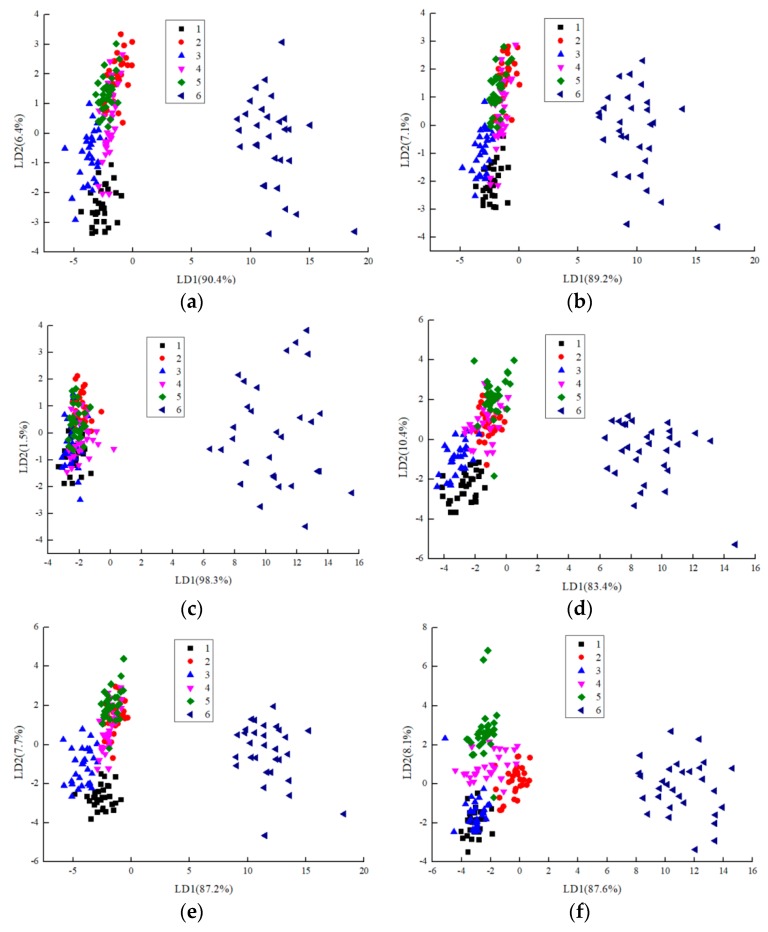
Linear discriminant analysis (LDA) results for different characteristic values (integral value (**a**); wavelet energy value (**b**); maximum gradient value (**c**); average differential value (**d**); relation steady-state response average value (**e**); and variance value (**f**)).

Linear discriminant analysis is a statistical method that can determine which group the sample belongs. This method maximizes the variance between categories and minimizes the variance within categories. Figure 4 shows the linear discriminant analysis results for different feature vectors on fish meal samples of different qualities. As shown in the figure, the results of linear discriminant analysis were distinctly better than those of principal component analysis. PCA only fits a handful of samples at a time. If the number of fish meal samples to be investigated increases, the fish meal samples with different storage times will become crowded and not easily distinguished in the PCA chart. The distribution structures of all linear discriminant analyses were particularly similar, due to similar data structures. Although fresh samples were not easily distinguished because the first two discriminant functions were applied in the linear discriminant analysis chart, through the discriminant analysis results of the five discriminant functions. The accuracy rate of the linear discriminant analysis, using integral values as feature vectors, was 90%, and the accuracy rate of linear discriminant analysis, using the wavelet energy values as feature vectors was 85.6%, and the accuracy rate of linear discriminant analysis, using the maximum gradient values as feature vectors was 56.7%. The correct rate of linear discriminant analysis using the average differential value as feature vector was 89.4%, the correct rate of linear discriminant analysis using the relation steady-state response average value as feature vector was 93.9%, and the correct rate of linear discriminant analysis using the variance value as feature vector was 92.8%. Therefore, the accuracy rate using the relation steady-state response average value to evaluate fish meal samples with different qualities was the highest, and the accuracy rate using the maximum gradient value to analyze fish meal samples with different qualities was the lowest.

### 3.5. Accuracy Comparison of Single Feature Classification under Different Classification Methods

To determine which features contribute most to the classification of fish meal quality, 21 fish meal samples of different storage time grades of each group were randomly selected as training sets (total of 126 samples) to train fish meal samples with different qualities, and the remaining 9 samples (total of 54 samples) of each group were employed as prediction sets. The integral value (INV), wavelet energy value (WEV), maximum gradient value (MGV), average differential value (ADV), relative steady-state response average value (RSAV), and variance value (VARV) were employed as characteristic parameters to model. With 10 sensor response data as the input, and fish meal samples at different storage time levels as the output, the classification accuracy of various eigenvalue was tested using a multi-layer perceptron neural network classification method, random forest classification method, K-nearest neighbor algorithm (K = 3), support vector machine algorithm, Naïve Bayes classification method, and five recognition modes. The test results were listed in Table 5.

As shown in Table 5, the highest accuracy rate was obtained using the random forest classification algorithm. The lowest classification accuracy rate was obtained using the support vector machine algorithm. This finding may be attributed to the fact that the RF classification method [42] is an integrated learning algorithm that consists of multiple decision trees (DTs). The algorithm is suitable for processing high-dimensional data and runs relatively fast. Its execution process is described as follows: First, the N-group training set is extracted from the original data; second, the N-group training set is used to construct N decision trees. During the growth process of each tree, m (m < M) characteristic variables are randomly selected from all M characteristic variables to divide the internal nodes. Last, the prediction results of N decision trees are collected and the category of new samples is determined by voting.

For the MLPNN (multilayer perceptron neural network) classifier, the highest accuracy of discriminating different quality fish meal samples was obtained using the relation steady-state response average value as a feature vector, and the lowest accuracy of discriminating different quality fish meal samples was obtained using the maximum gradient value as a feature vector. For the RF classifier, the highest accuracy was obtained using the integral value and the wavelet energy value, as features to discriminate the different quality fish meal samples, and the lowest accuracy was obtained using the maximum gradient value as an eigenvector to discriminate different quality fish meal samples. For the KNN classifier, the highest accuracy of discriminating fish meal samples, with different qualities, was obtained using the relation steady-state response average value as a feature vector, and the lowest accuracy of discriminating fish meal samples with different qualities was obtained using the maximum gradient value, as a feature vector. For the SVM classifier, the highest accuracy was obtained using the integral value as a feature vector to discriminate different quality fish meal samples, and the lowest accuracy was obtained using the variance value as a feature vector to discriminate different quality fish meal samples. For the Naive Bayes classifier, the highest accuracy of discriminating fish meal samples with different quality was obtained using the average differential value and relation steady-state response average value as eigenvectors, and the lowest accuracy of discriminating fish meal samples with different qualities was obtained using the maximum gradient value as eigenvectors. Therefore, for the accuracy of single feature classification, the highest accuracy was obtained using the integral value, relation steady-state response average value and average differential value as eigenvectors to discriminate different quality fish meal samples, and the lowest accuracy was obtained using the maximum gradient value as eigenvector to discriminate different quality fish meal samples. These findings were almost identical to the results of the previously mentioned PCA and LDA analysis. The similarity may be attributed to the observation that the test response signal of the electronic nose to the sample is relatively stable. The feature points extracted by the maximum gradient method of the response curve of a sample will be observed at different times of the response curve, which is worse than the analysis of the relation steady-state response average value, while the relation steady-state response average value and integral value method can extract the stable signal of the electronic nose signal, so the prediction results are better.

## 4. Conclusions

In this paper, the response curves of the fish meal samples, with different storage times, were obtained using a self-made electronic nose. Most VOCS emitted from fish meal malodor are reducing gases, and the fingerprints of the fish meal with different qualities differed, which enabled the electronic nose to distinguish between different quality fish meal.

The discriminant result of the linear discriminant analysis was better than those of the principal component analysis. In this paper, the principal component analysis and linear discriminant analysis of fish meal quality were performed by selecting six features: Integral value, wavelet energy value, maximum gradient value, average differential value, relation steady-state response average value, and variance value. By PCA and LDA analysis, the sum of the first principal component analysis value and the second principal component analysis value exceeded 85% when all features were considered to be parameters. The effect of using the relation steady-state response average value as the characteristic parameter was better, and the accuracy of linear discriminant analysis was 93.9%. The effect of using the maximum gradient value as the characteristic parameter was worst, and the correct rate of linear discriminant analysis was only 56.7%.

To further determine the best and worst eigenvalues in fish meal quality detection, the classification methods of a multi-layer perceptron neural network, random forest classification, K-nearest neighbor algorithm, support vector machine algorithm, Bayesian classification method and five recognition modes were used to classify the fish meal quality, and the highest accuracy was obtained using the random forest classification algorithm. The highest accuracy was obtained using the integral value, relation steady-state response average value, and average differential value as eigenvectors, and the lowest accuracy was obtained using the maximum gradient value as eigenvectors.

The results indicated that the electronic nose could classify the fish meal with different qualities. When selecting an optimal feature, the classification effect was better. Further research shows that the electronic nose has strong sensitivity and selectivity in detecting fish meal samples of different storage times.

## Figures and Tables

**Table 1:** The acid value of each grade sample.

Samples	1	2	3	4	5	6
Acid Value	2.99	3.78	4.63	5.4	6.58	9.25

**Table 2:** Gas sensor array and its properties.

Sensor Numbers	Sensor Types	Sensitive Substances	Detection Ranges
S1	TGS822	Alcohol, solvent vapors	50–5000 ppm
S2	TGS2602	General air contaminants	1–30 ppm
S3	TGS813	Carbon monoxide, ethanol, methane, hydrogen, isobutane	50–10,000 ppm
S4	TGS2620	Solvent vapours	50–5000 ppm
S5	MQ136	H_2_S	1–200 ppm
S6	TGS2600	Carbon monoxide, hydrogen	1–30 ppm
S7	MQ139	R11, R22, R113, R134A, halogen	10–1000 ppm
S8	TGS2610	Ethanol, hydrogen, methane, isobutane/propane	500–10,000 ppm
S9	MQ137	Ammonia and amine compounds	5–500 ppm
S10	TGS2611	Ethanol, hydrogen, isobutane, methane	500–10,000 ppm

**Table 3:** The Repeatability of response values of E-nose 10 sensors.

Sensor	S1	S2	S3	S4	S5	S6	S7	S8	S9	S10
RSD/%	2.72	1.05	3.15	4.65	2.43	3.53	1.91	2.24	1.26	2.27

**Table 4:** The identified volatile flavor compounds of fish meal headspace using SPME-GC-MS.

No.	Retention Time	Molecular Formula	Volatile Compounds	RI		Identification
				Calculated Value	Literature Value	
1	2.45	C_3_H_9_N	N,N-dimethyl-methylamine	-	502	MS, RI
2	3.4	C_5_H_10_O	Pentanal	-	699	MS, RI
3	4.61	C_3_H_6_O_2_	Propanoic acid	700	700	MS, RI
4	5.35	C_5_H_10_O	(Z)-2-penten-1-ol	767	767	MS, RI
5	6.31	C_6_H_12_O	Hexanal	800	800	MS, RI
6	7.65	C_4_H_8_O_2_	Butanoic acid	832	805	MS, RI
7	8.44	C_6_H_10_O	(E) -2-hexenal	851	854	MS, RI
8	9.28	C_5_H_10_O_2_	3-Methyl-butanoic acid	871	863	MS, RI
9	9.71	C_5_H_10_O_2_	2-Methyl-butanoic acid	882	861	MS, RI
10	10.47	C_7_H_14_O	Heptanal	900	901	MS, RI
11	11.19	C_8_H_9_NO_2_	Methoxy-phenyl-oxime	918	-	MS
12	12.89	C_7_H_6_O	Benzaldehyde	958	962	MS, RI
13	13.47	C_6_H_12_O_2_	4-Methyl-pentanoic acid	972	949	MS, RI
14	13.83	C_8_H_16_O	1-Octen-3-ol	980	980	MS, RI
15	14.14	C_8_H_9_NO_2_	Methyl-carbamic acid phenyl ester	987	-	MS
16	14.54	C_6_H_12_O_2_	Hexanoic acid	997	990	MS, RI
17	14.65	C_7_H_10_N_2_	2-ethyl-5-methyl-pyrazine	1000	1005	MS, RI
18	14.75	C_8_H_16_O	octanal	1002	1003	MS, RI
19	15.73	C_8_H_16_O_3_	3-Hydroxy-2,2-dimethyl-butanoic acid ethyl ester	1027	-	MS
20	16.11	C_7_H_8_O	Benzyl alcohol	1036	1036	MS, RI
21	16.34	C_8_H_8_O	Benzeneacetaldehyde	1042	1045	MS, RI
22	16.84	C_6_H_10_O_2_	5-Ethyldihydro-2(3H)-furanone	1054	1057	MS, RI
23	18.79	C_9_H_18_O	Nonanal	1103	1104	MS, RI
24	21.46	C_5_H_9_NO	2-Piperidinone	1175	1174	MS, RI
25	21.67	C_10_H_8_	Naphthalene	1181	1182	MS, RI
26	22.1	C_10_H_20_O	2-Decanone	1192	1193	MS, RI
27	22.56	C_10_H_20_O	Decanal	1205	1206	MS, RI
28	25.67	C_11_H_22_O	2-Undecanone	1293	1294	MS, RI
29	26.46	C_11_H_18_N_2_	2,5-Dimethyl-3-(3-methyl-butyl) pyrazine	1320	1315	MS, RI
30	28.58	C_14_H_30_	Tetradecane	1400	1400	MS, RI
31	30.47	C_15_H_24_O	Butylated hydroxytoluene	1519	1513	MS, RI
32	34.78	C_16_H_32_O_2_	n- Hexadecanoic acid	1960	1968	MS, RI

**Table 5:** Accuracy comparison of single feature classification for different classification methods.

Algorithm	MLPNN		RF		KNN		SVM		Naive Bayes	
	Training	Validation	Training	Validation	Training	Validation	Training	Validation	Training	Validation
INV	91	91.4	100	90.7	90.5	74.1	84.09	70.83	90.5	77.8
WEV	96.7	84.5	100	90.7	87.3	64.8	78.03	68.75	87.3	83.3
MGV	72.1	72.4	100	83.3	83.3	61.1	62.12	54.17	81	70.4
ADV	91	91.4	100	85.2	92.9	70.4	78.03	56.25	89.7	88.9
RSAV	93.4	91.4	100	87	92.1	81.5	78.79	62.5	88.1	87
VARV	82	82.8	100	88.9	90.5	88.9	56.06	35.42	84.1	77.8
